# Integration and Causality in Enactive Approaches to Psychiatry

**DOI:** 10.3389/fpsyt.2022.870122

**Published:** 2022-07-04

**Authors:** Shaun Gallagher

**Affiliations:** ^1^Department of Philosophy, University of Memphis, Memphis, TN, United States; ^2^School of Liberal Arts, University of Wollongong, Wollongong, NSW, Australia

**Keywords:** integration, psychiatry, autism spectrum disorder, causality, levels

## Abstract

In this paper I address what has been called the integration problem in psychiatry. This problem is tied to conceptions of causality and explanatory levels in our understanding of mind. I take an interdisciplinary enactive perspective to develop a 3-fold method for exploring the dynamics of integration, based on a concept of dynamical causation and a non-hierarchical (level-free) notion of gestalt. I also consider Autism Spectrum Disorder (ASD) as a test case.

## The Integration Problem in Psychiatry

The integration problem concerns how to understand the coupling or interaction among all of the diverse processes that may be involved in psychiatric disorders (1–4). The processes that need to be integrated, for example, genetic, neuronal, psychological, social and cultural processes, are commonly said to be on different levels or involve different scales. Integrative accounts of psychiatric disorders can be narrow or wide, depending on the range of processes or factors included. For example, Gerrans (2), in his book on delusions, proposes a relatively narrow account of the “cognitive architecture” of delusions by integrating neuronal and phenomenological elements, with narrative generated in the brain's default mode network^1^. Gerrans, treating the cognitive system as an information processing one, invokes hierarchical arrangements of (top-down) predictive processing (requiring reference states for comparison between higher and lower levels), combined with a mainly bottom-up neural network theory. He also pursues an explanatory strategy in terms of causal relevance, appealing to new mechanist models (5), and Woodward's (6) interventionist view of causality. One version of the integration problem, then, is to understand how to adjudicate among multiple explanations or disciplines [see (7) for a recent attempt to address this problem].

The challenge for Gerran's account is precisely how to integrate these different models—all of which depend on the concept of levels. To be clear, the issues is not just about integrating cognitive/narrative, personal/experiential, and sub-personal/neurological levels, but also integrating the different conceptions of levels involved in the various theories and models to which Gerrans appeals. He admits this is a problem.

The notion of levels is ubiquitous, but not everyone uses it in the same way. It can refer to ordering relationships between theories…; the objects of theories ordered by size or complexity, e.g., cells are smaller and less complex than the organs they make up; functional analyzes, e.g., vision is a higher-level property than edge detection; or levels of mereological containment, e.g., parts are at a lower level than wholes. These uses can overlap. The notion of level relevant to [Gerrran's analysis] is that of mechanism. Higher levels correspond to the organization of components, lower levels to components [(2), p. 229–230].

The mechanist view, however, where levels are mereologically defined, may not match up with hierarchical arrangements defined by predictive processing, which specifies reference state comparators between higher and lower levels.

Another example can be found in phenomenological psychiatry. Sass et al. (4), drawing on both phenomenology and Engel's (9) biopsychosocial model, proposes a bio-pheno-social integrative account: Bio (genetics; neural) + phenomenology + social factors. Sass's account involves a wide integration insofar as he includes social factors. Sass worries about how to explain the relations among the various “component processes.” Are they arranged in a hierarchical fashion; are they causal or constitutive? Sass finds his analysis entangled in the theoretical framework of levels, and he remains pessimistic.

[These] questions pertain to theoretical issues that are difficult or even impossible to settle, such as overall attitudes toward holistic explanation or the enigmatic mind/body problem (generally viewed as utterly insoluble)” [(4), p. 725]

The proliferation of different levels and different kinds of levels has motivated even theorists who often employ the notion of levels to add some qualifications and to move away from the concept of hierarchy, especially in the context of understanding psychiatric disorders. Eronen [(10), p. 929], for example, makes this point in the context of psychopathology.

[D]ifferences in time scales could be used to define levels, but scales are continuous, where the boundary between levels should be drawn may not have a clear answer…. The upshot is that defining levels in psychopathology is far from straightforward. The exact levels and their significance can vary strongly from context to context. In psychology, it is often unclear how part-whole or scale-based levels should be understood in the first place. This suggests that levels in psychopathology are best seen as heuristic idealizations that are helpful in making rough distinctions, but do not mark deep ontological features of the world.

It's possible to develop explanations that are not based on hierarchical levels. Bechtel (11), for instance, develops a mechanist view that favors a heterarchical set of neural control mechanisms, rejecting a hierarchical organization in favor of lateral constraints within a network that “does not impose any requirement of hierarchical organization” [(11), p. 31]. Likewise, Woodward (12), commenting on Eronen's “levels eliminativism”—“the thought that we would be better off avoiding level talk entirely”—suggests it is motivated by the fact that researchers operate with different conceptions of levels without always distinguishing them. Woodward, however, endorses the concept of levels suggesting it continues to do some useful work in science. After discussing several different types of levels, he focuses on an interactionist conception, closely tied to the role of different spatial, temporal, or energetic scales in constructing causal models. Different scales matter specifically as they relate to the “strength and nature” of causal interactions. In this respect, it's clear that differences in scales are doing some of the explanatory work. As he puts it, “sometimes when nature is kind” we have relatively clean distinctions between scales, “so that what happens at one length of energy scale can be understood largely independently of what happens at other scales, and this in turn leads us to think of interactions at one scale as at a different level than interactions at other scales” [(12), p. 429]. The concept of scales, however, is different from the concept of levels. Craver and Darden [(13), p. 162ff], for example, assume this distinction in their discussion of integration. They distinguish between levels of products, levels of units, levels of causation, levels of composition, levels of mechanisms, etc. Differences in time scales, in contrast, involve differences in how we measure processes.

The ideas of a heterarchical set of causal relations, involving reciprocal control processes, framed in terms of different temporal and spatial scales could take up some of the explanatory slack if we were to give up hierarchical level explanations. In contrast to hierarchical level-based explanations in psychiatry, de Haan (1) offers an alternative enactive model of wide integration based on what she calls “organizational causality.” Not unlike the bio-pheno-social model, it includes phenomenological, social and neurobiological processes. De Haan importantly adds an “existential” dimension—the idea that the person is reflectively aware of her own situation and can take an evaluative attitude toward it. She argues that discussions of reciprocal or circular causality found in some enactivist models (14–16), to the extent that they still depend on the notion of levels, continue to present the temptation of reductionism. Moreover, questions about how one level is causally related to another tend to frame the analysis in dyadic rather than holistic terms. For example, Fuchs writes: “mental illnesses are marked by a disruption of vertical circular causality; that is, the interplay between lower-level processes and higher faculties of the organism” [(14), p. 331]. On this view, circular causality just is a form of reciprocal, downward and upward relations between parts and whole [(15); also see (17)]. In a psychiatric context, although more than two elements may be involved in a more complex pattern of causality that can be circular, the pattern can also involve multiple, non-linear or indirect interactions across different time scales. De Haan argues organizational causality allows us to move away from the idea of levels—so causal relations are neither bottom-up nor top-down; rather, they can involve a heterarchical set of relations organized more holistically.

What is organizational causality? Instead of involving levels or hierarchical arrangements, de Haan associates organizational causality with the idea of emergence as fusion. On this point De Haan follows Humphreys (18): “when emergence occurs the lower-level property instances go out of existence in producing the higher-level emergent instances.” To explain emergence as fusion de Haan offers the analogy of a cake, where all of the ingredients, even if they start out as differentiated, once baked, become fused in such a way that one can no longer distinguish sugar from flour from flavorings, etc. This is, of course, a cake without layers. I agree that the right conception of causality can lead to a better solution than the ambiguous mix of levels noted in the theories discussed above. The concept of fusion, however, is unhelpful in the psychiatric context since, even if we bracket the talk of “levels,” as de Haan wants to do, the various processes or factors that constitute the psychiatric context do not go out of existence, nor do they fuse to the point where we cannot discriminate among processes that are clearly affective or clearly cognitive, or clearly social, etc. even if such processes are dynamically integrated. In psychiatric practice we need a model of integration that does not subvert useful distinctions among the neurobiological, the phenomenological, the social and the existential, to use de Haan's own list of perspectives.

De Haan indicates that the point she wants to defend is that “we can no longer assume that parts or processes still have the same properties once they have become part of a whole” (private correspondence). As she puts it in her book: “the parts or processes prior to this configuration are thus not the same as the parts or processes after this configuration: their relational structure carves out different “abilities” and “inabilities” than they had before, either in isolation or as part of a different constellation” (p. 116). I agree, but the notion of fusion does not capture this idea. With the idea of fusion, we go from a confused mishmash of levels (as noted by Gerrans), to a situation in which, not only levels, but all useful distinctions disappear.^2^

This still leaves the problem of integration—the problem of understanding how the diverse processes involved in our everyday existence, that can go awry in psychiatric disorders, are related or coupled. What we need is a solution that eschews the idea of levels, and the accompanying temptation of reductionism [see (10, 19)], but still leaves intact important distinctions among different contributing factors. In terms of the etiology of psychiatric disorders, for example, it is not clear that neuronal processes are necessarily more basic than social processes, or that affective processes are at a lower level than cognitive processes. Yet we still need to discriminate affect from cognitive processes, and neuronal from social processes, even if we do not want to think of them as operating on different levels, and even if they are in some way causally integrated or meshed or transformed. Even if an individual's affective life changes by means of narrative therapy, for example, neither affect nor narrative go out of existence to form something else. We can acknowledge with de Haan that processes involved in psychiatric disorders change as they mesh with other changing processes, but this is not a fusion into indistinguishable processes. Indeed, when de Haan discusses psychiatric diagnosis and treatment, she reverts to the concept of complex patterns, framed in terms of network models [(1), p. 244ff].

Although de Haan's term “organizational causality” works well here, to avoid the idea of emergence as fusion I'll refer to the idea of *dynamical causality*, where in some cases relations are non-linear, and transformational, but lead to neither hierarchical arrangements nor fusion. Instead of fusion, we can think of processes that involve dynamical transformations. When one factor comes into a dynamical causal relation with another factor, both factors may be transformed or changed, but they do not disappear in the process. This describes a dynamical gestalt—a set of distinguishable processes that are dynamically related, but are not defined as on different levels. Although one usually thinks of a gestalt as involving a whole that is more than the sum of its parts, a dynamical gestalt, rather than constituted in part-whole relations, is constituted in the dynamical causal relations among the processes forming a particular pattern. In the case of dynamical causality an intervention above a certain threshold may result in changes to one or more factors, directly or indirectly, serially or in parallel, and include looping effects, all of which depend on parameters of flexibility in the gestalt relations.

## A 3-Part Method to This Madness

If the integration problem is not solved in Gerrans' (narrow) or Sass's (wide) hierarchical level model, and if we have reasons to doubt that de Haan's fusion model will work in this context, then the idea of a dynamical gestalt offers an alternative way of addressing the integration problem. For purposes of characterizing a gestalt, it is not enough to simply list the relevant factors or processes; rather, we need to say specifically how all of the factors relate (20–22). To be clear, the idea that there are dynamical connections among the various factors and processes of the gestalt is part of its definition. In this regard, the concept of gestalt at stake comes close to Kelso's (23) notion of dynamical pattern. The relevant notion of causality is non-linear/dynamical causality rather than reciprocal, circular, or organizational causality (understood in terms of fusion). The question, however, is what this means in the context of psychiatry.

This idea is not entirely new. The concept of gestalt has played an important role in phenomenological psychiatry. Thus, for example, Jaspers states:

All research differentiates, separates and studies individual particulars in which it tries to discover certain general laws. Yet all these individual particulars are taken out from what is in reality a complex unity. In grasping particulars, we make a mistake if we forget the comprehensive whole in which and through which they exist. This never becomes the direct object of our study, but only does so *via* the particulars. […] We can state the following in relation to it: the whole comes before its parts; the whole is not the sum of its parts… it is form [(24), p. 28–29. 504].

In this context, the notion of gestalt involves relational criteria that help to capture psychiatric disorders, which tend to be multifactorial. To the extent that we think of such disorders as a gestalt arrangement of factors, each individual case of a particular disorder may show different patterns of dynamical relations among the various factors. Different factors may involve different weights in different individuals (which can be measured in a dynamical analysis, see Section Coordination Dynamics). For example, anorexia will present differently in a disciplined person with high degrees of self-control than in an impulsive individual [(25), p. 334]. Variations in the amount of social support or different cultural contexts may determine how a disorder develops. But we can expect to see typical patterns associated with different disorders. Schizophrenia, for example, may affect multiple aspects of experience, cognition, mood and agency in somewhat typical ways (26), while panic disorder will be more aspect limited in its impact (27).

More generally, as Sass et al. [(28), p. 12] suggest, in regard to characterizing the elusive aspects of changes in psychopathology, “mutations of worldly experience (like mutations of ipseity or basic self-experience) typically have an overall or holistic character that defies ready operationalization into distinct features or factors.” Sass's “bio-pheno-social” model attempts to capture the multifactorial complexity of such disturbances. Thus, “alterations at the level of the lived-body [have] profound implications for both interpersonal and intersubjective dimensions of existence,” and in schizophrenia, “environmental/social stressors, including childhood trauma and abuse, social defeat, and cultural dislocation/alienation” may play some pathogenetic role [(4), p. 722].

Rather than positing a hierarchical relation that treats ipseity as more basic, and then working outward or upward to more secondary factors, however, the notion of dynamical gestalt stays with the proviso mentioned above, that disorders “typically have an overall or holistic character that defies ready operationalization into distinct features or factors.” Perhaps, then, the alternative worry then would be, as Parnas and Henriksen (29) express it, that “[t]his gestalt is intrinsically elusive and resists any simple, straightforward attempt to define it.”

To deal with this worry, consider a clue that we can find in Gerrans's analysis. In discussing loss of the sense of agency in some schizophrenic delusions, he references studies of expert performance where, on some views, expert performance involves the absence of a sense of agency as one is on automatic pilot, or “in the flow,” compared to a strong sense of agency in novice performance where there is top-down cognitive control. “We *are* more aware of our agency when learning a musical instrument … than when performing a task automatically and successfully” [(2), p. 169]. Although on this point Gerrans would seemingly endorse the Dreyfus model of expert performance (30), where expert performance/embodied coping is a mindless (non-representational) being-in-the-flow, most of Gerrans analysis puts him closer to theorists who argue, against Dreyfus, that performance involves mindful elements. In this regard, they conceive of a top-down process where low-order automatic processes of embodied coping are modulated by higher-order, reflective (representational) cognitive aspects.

This debate in the area of performance studies has motivated the development of a model that can serve as a beginning point for analyzing the factors and relations in a dynamical gestalt—the meshed architecture model (31). It's important to note that the use of this model is a first step rather than a finished product. It's meant to be a heuristic to help map out the relevant processes and to set up the next two methodological steps. More specifically, I propose the use of an enhanced meshed architecture model as the first part of a 3-fold method for explaining the notion of dynamical gestalt, and solving the integration problem. A second part builds on interventionist conceptions of causality (6). And the third part employs Kelso's coordination dynamics approach (32).

### An Enhanced Meshed Architecture

The idea of a dynamical gestalt means that the various processes and factors involved are dynamically related to form a whole. This holistic view motivates a number of philosophical issues, but practically, for psychiatry, it presents a challenge both in regard to diagnosis and therapeutic interventions. Ideally, one might want to say precisely what process or factor, or what set of processes or factors, in any particular case, is problematic, and how it might be addressed. Realistically, it is difficult to be precise in this regard. The model of a meshed architecture, however, can help to map out which factors are relevant, and how they are related. I take this to be a first step in a three-step approach to gaining an explanatory grasp of specific disorders.

To explain the notion of the meshed architecture, I'm going to short-circuit all the debates in the performance literature (concerning dance, musical performance, and acting) and simply present an enhanced version of the “meshed architecture model,” which I understand to be consistent with an enactive approach that can generalize to explanations of embodied-situated-social cognition (33), and some psychiatric disorders such as depression and schizophrenia, as well as conditions like autism spectrum disorder.

In performance studies, one challenge is to explain how complex cognitive processes, such as memory and attention, can guide or control what are construed as automatic motor processes in skilled performance. In many cases performance is fast and seemingly automatic, yet it is also context-sensitive and strategic in a way that suggests there is an “interpenetration of thought and action” [(34), p. 80]. In contrast to Dreyfus' notion that skilled performance is mindless, Christensen et al. (31) propose the meshed architecture model which involves an integration of cognitive and bodily (sensorimotor) processes. The mesh involves “a broadly hierarchical division of control responsibilities, with cognitive control usually focused on strategic aspects of performance and automatic [body-schematic] processes more concerned with implementation” [(31), p. 43]. On this view, cognitive control “counteracts automaticity” and introduces flexibility into motor control. Admittedly, this model starts out endorsing a hierarchical arrangement. Indeed, one pictures a vertical hierarchy that divides into two poles: cognitive at the top, descending to do its job; automatic bodily processes at the bottom receiving instructions when necessary. This initial model of the meshed architecture is, I've argued, too top-down and overly intellectualized (33, 35), and it involves a concept of hierarchical levels that we are trying to avoid, I'll suggest ways to move beyond talking about such levels in the following enhanced model.

A more complex, enhanced conception of the meshed architecture includes three aspects that are not accounted for in the original model: (1) *intrinsic control*—integration processes that work from the bottom-up; (2) an important role for *affectivity*; and (3) a complexity introduced by what we can call a *horizontal axis*. The structure defined by mapping elements on vertical and horizontal axes operates as a heuristic that we can then drop in favor of a more dynamical, non-hierarchical, holistic model.

1. A concept of *intrinsic control*: control is not entirely top-down, but rather, on the vertical axis there are important bottom-up processes that are not automatic.

Motor control, body-schematic processes are attuned by practice and provide the freedom to pay mindful attention to relevant surrounding factors. But such processes should not be viewed as fully automatic. Jonides et al. (36), for example, explicitly argue that motor control processes overall do not automate. Evidence from kinematics suggests that body-schematic processes are perfectly specific, adaptive and highly dynamical such that they adaptively attune to differences in situations (environmental conditions, object positions, initial and unfolding body postures) and agentive intentions. In contrast to an intellectualist view that insists on the automaticity and “perfectly general” nature of such processes [e.g., (37)], body-schematic processes are neither fully automatic (blindly pre-set, like a reflex, to do the same thing in each circumstance, regardless of differences), such that in varying circumstances they require top-down cognitive guidance, nor perfectly general; they rather include a specificity that involves an “enormous number (which often reaches three figures) of degrees of freedom” (38), as well as a complex temporal organization involving anticipatory processes across skeletal geometry, kinematic phase constraints, muscular geometry, and the dynamics that characterize the relationship between kinematics and geometry (39, 40). These complex processes come to intelligently align with a particular intentional trajectory, *not automatically* (clicking into place in a machine-like fashion), but rather, in a way that is flexibily attuned to the particularities of the situation.

We can think of this attunement as a form of habit, developed when the body “acquires the power of responding with a certain type of solution to a certain form of situation” [(41), p. 143]. Habit involves an intelligent response, where intelligence is built into the movement. Instead of blind automatic repetition, habit is an open and adaptive way in which the body learns to cope with familiar or unfamiliar situations. Intrinsic control involves motoric processes that are already context-sensitive, smart, open and adaptive, such that they can even elicit or shape or enable the required cognitive elements that may be contingently incorporated into the mesh. Sensorimotor processes regulate the activation of specific cognitive processes when they are needed. Accordingly, mindfulness is not simply imported from the “top;” it's already built into the “bottom,” and, again in some cases, such habitual processes may be what guide any need for more reflective cognitive processes.

Skills and habits can persist even in pathologies where forms of memory that are more cognitive are lost, as in cases of dementia. Christian Tewes cites a patient of Thomas Fuchs who in advanced stages of dementia is still able to skillfully play football with his grandchildren [(42), p. 301]. As Tewes explains, this is not automatic behavior but “situation-specific embedded action patterns that depend on attention to and implicit understanding or know-how of the respective social context” [(43), p. 383]. Such skill is also strongly linked to previous life experiences even if, as in the case of dementia, these life experiences cannot be explicitly recalled by the subject.

2. *Affective processes* modulate the dynamics of integration.

In addition to the reciprocal vertical integration of cognition and body-schematic attunement, affectivity is an important factor. In the broadest sense this includes emotion processes, but also more general and basic bodily states such as hunger/satiation, fatigue/high energy, pain/no pain/pleasure, including “existential feelings” (44), and what Maise (45) calls “affective framing,” which shapes our ability to cope with the surrounding world (46). Along with skills and habits, affect introduces possible modulations of functional integration with that world. Affect may work differently in different types of skilled actions, where important differences may have to do with the way that affective factors are integrated with motoric/agentive factors, the kinetic and kinaesthetic feelings associated with body-schematic processes, and how all of these processes integrate with environmental constraints and affordances (47, 48).

Affective processes can directly shape body-schematic processes—slowing down or speeding up such processes, for example, or leading to the adoption of certain initial postures that may influence how agents are functionally integrated with the world. Affect and body-schematic processes are integrated, but affect also allows for an integration attuned to targets and environmental features located on a horizontal axis.

3. A *horizontal axis* that integrates ecological, social and cultural-normative factors.

**Figure 1:**
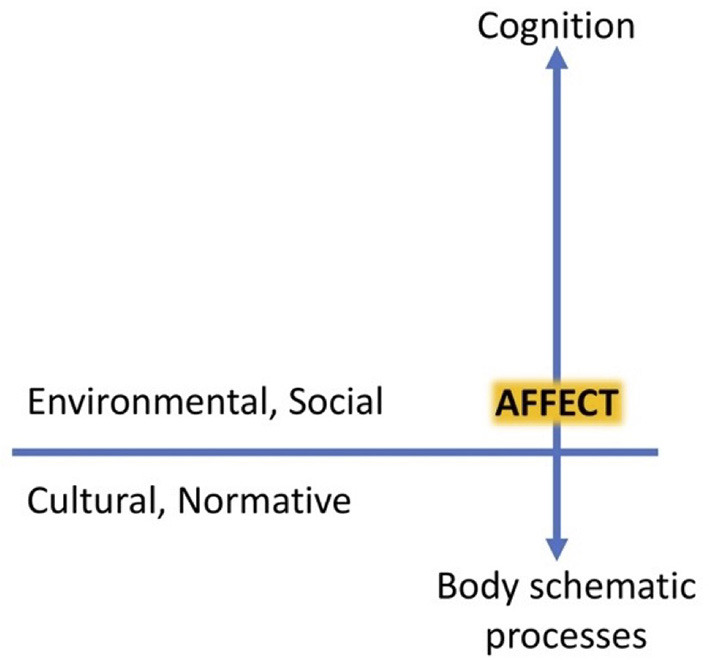
Vertical and horizontal axes of the meshed architecture [from Gallagher et al. (49)].

The more enhanced conception of the meshed architecture incorporates horizontal integration of ecological, social, cultural/normative aspects, including physical and social affordances (see Figure 1). On this view, what makes performance what it is not entirely internal to the performer. To return to Gerrans' comment on the sense of agency, as one engages in a particular action, one's sense of agency may be modulated (causally influenced) by affect, but also by social context and the quality and quantity of affordances available. When, for example, the performer “can ‘feel' that her motor system has the right configuration” (31), this configuration is just the right one to mesh with the specifics of the performer's physical and social environment. Neither body-schematic processes nor affective processes are isolated from the agent's environment; rather they are attuned to both stabilities and variations in environmental factors, including other agents. The subject's experiences are situated, i.e., functionally integrated with the environment, which is not only physical, but also socially, culturally, and normatively defined.

The notion of a meshed architecture offers a pragmatic heuristic in the service of mapping or diagraming relations. On the one hand, the distinction between vertical and horizontal axes in the meshed architecture offers a way to start thinking about how the different processes and factors integrate or mesh. On the other hand, thinking about how different factors integrate as a dynamical gestalt implies a holistic rather than a hierarchical arrangement of factors. As we've seen, some integrative approaches remain narrow in the sense that all of the important processes are conceived of as happening “in the head,” or specifically on the vertical axis. For example, Gerrans (2) explains the “cognitive architecture” of schizophrenic delusions of control and thought insertion by integrating elements that are neuronal and phenomenological (e.g., the sense of agency), with cognitive-narrative components generated in the default mode network. These are all conceived to be arranged in top-down/bottom-up inferential processes on the vertical axis. As he indicates, “the system is hierarchical, with each level in the hierarchy using the predictive coding strategy. Error systems propagate up and down the hierarchy” (p. 165). One's sense of agency depends on such processes. It arises in the interactions between body-schematic/proprioceptive feedback loops and “higher-level, explicit, visually guided control, or (sometimes) mental rehearsal of action' (p. 166). If it goes missing in schizophrenic delusional experiences, that can be explained by disruptions in such processes. Gerrans' analysis gets more complex when he attempts to explain why the schizophrenic attributes the action or thought to an external agent. Indeed, issues surrounding the sense of agency are even more complex when one takes into account the role of ecological and social factors (50). One needs to go wider and to incorporate the horizontal axis into the explanation. It's not clear, however, where social factors fit into the hierarchy that Gerrans describes. Moreover, on a dynamical gestalt view it's not clear why we should think of them as higher-order processes compared to neuronal or body-schematic/proprioceptive processes (unless we have already made some reductionist assumptions or have decided apriori that relations have to be hierarchically arranged). Neural and social processes can be co-temporaneous and either aligned or integrated across time scales in non-linear arrangements involving dynamical causality, without an up or down.

For the vast variety of psychopathological phenomena, including delusions, we need to go wider and to scale out to include the horizontal axis—the ecological, social, cultural, and normative factors. If we think of the mesh as a dynamical, constantly changing gestalt, we may still be able to get a bearing on what factors might be heavily weighted and duly or unduly influential in any particular case. But further explanation requires an account of the dynamical relations that exist amongst these elements so that we start to think of the mesh as a set of processes in which vertical and horizontal are clearly just explanatory abstractions. To be able to specify these precise relations, however, we require two other parts of the method.

### Interventionist Causality

The second part of this methodological approach derives from Woodward's (6) conception of interventionist causality in contexts of both experimentation and therapy. Woodward understands causality to be a probabilistic counterfactual dependence relation. In other words, X causally relates to Y iff an intervention on X makes a difference to the probability of Y assuming appropriate background conditions. Causal relations are established using interventions to show probabilistic dependencies between variables. Accordingly, this theory allows for causal relations between a wide variety of factors (biological, psychological, social, etc.,) regardless of whether or not one thinks of such factors as existing on different levels. We can take intervention in a broad sense to characterize any possible event or change that could influence a particular factor or process. This would include experimental and therapeutic interventions. In a gestalt arrangement, a change to one variable, above a certain threshold, will likely cause changes in other variables in the system. That includes any change in brain, organism, environment, or in any particular habit or practice. These may be changes in a life circumstance (for example, a traumatic experience), or in a controlled experiment (for example, the presentation of stimuli that would alter a person's mood), or in a therapeutic context (for example, medicating or changing cognitive or motoric habits). Life, or the experimenter, or the therapist intervenes on a particular process. Above a certain threshold, we would expect that such an intervention could have significant effects on the processes or factors that define the dynamical gestalt.

If we want to understand how changes in one factor can lead to changes in other factors, or to more holistic changes, we can devise experiments that precisely target one particular variable to see what other processes might change as the result. This can be more than just an intervention on an internal mechanism (e.g., a drug effect on neuronal processes)—it can be an intervention on a social factor, or a therapeutic intersubjective practice which can lead to a rearrangement of the entire gestalt [see, e.g., (51)]. Precision intervention methods allow us to identify the relations that are enabling, constraining, or that involve dynamical causality.

As an example, consider therapeutic interventions on body-schematic processes to address psychomotor retardation, deviant gait and posture in Major Depressive Disorders (MDD). In a meta-analysis Reed and Ones (52) reviewed 158 studies showing that positive affect is increased by engaging in physical exercise (e.g., walking). Studies have also shown that depression affects how self-related information is processed, biased toward the recall of affectively negative self-referent material and reduced processing of positive information. Michalak et al. (53, 54) conducted intervention experiments in which they caused alterations in posture or gait patterns. Such alterations led to changes in the subjects' recollections on self-referent positive vs. negative words, demonstrating that “biased memory toward self-referent negative material can be changed by manipulating [posture or] the style of walking” [(53), p. 124]. They thus conclude that “changes in the grossmotor system affect processes that are relevant in the etiology of depression…. Bodily aspects such as posture or movement patterns (e.g., gait characteristics) might be more than epiphenomena of psychopathology but also might contribute to an escalation of distorted processes in psychological disorders” [(54), p. 523; also see (55, 56), p. 146ff. for discussion].

Consider also that changes to ecological arrangements can result in changes to other factors. One can think of this broadly in terms of “cognitive niche construction” (57), where we design environments in ways that enhance our cognitive or affective processes, or in terms of the scaffolding function of designed environments where one can “manipulate the scaffolded by manipulating the scaffold” in order to make a difference (56). In therapeutic contexts manipulation of the environment may be facilitated by the use of virtual reality (VR). Specifically, the use of VR and mixed reality in clinical environments allows for the creation of (virtual) affordances to facilitate or support embodied, interactive, and affective therapies (58, 59). Psychotherapeutic applications of VR can address a variety of anxiety disorders (60, 61), and a variety of eating disorders such as Anorexia (62), as well as PTSD (63).

This interventionist logic can also be used to interpret a therapeutic experiment designed to understand capacities for social cognition in the case of ASD. Tager-Flusberg and Anderson (64), informed by the standard views of social-cognitive problems in autism as involving deficits in theory of mind (ToM), studied conversational ability in children with ASD. They hypothesized that impairment in conversational ability may be due to deficits in theory of mind (ToM) development. Hadwin et al. (65) then tested this hypothesis, training children with ASD on tasks that employ ToM (cognitive) abilities to understand mental states in others, focusing on beliefs, emotional states, and pretense. We can consider this an experimental test of a therapeutic intervention. Hadwin et al. compared conversation abilities prior- and post-training using four categories:

1. Simple answer—a child simply provides a one-word reply to a question and did not further engage in conversation
2. Developed answer—child is able to produce utterances that involved two or more sentences
3. Echolalic or repetitive answers
4. Unclear responses—non-sequiturs

After training on ToM tasks, the children did learn to pass tasks concerning emotional and belief understanding (but not pretense). They improved their ToM skills. However, there was no corresponding advance in social communication skills. Specifically, the experimenters found no improvement in the development of conversation and no increase in the use of mental state terms in speech [(65), p. 533]. The majority of children with ASD remained in the simple answer category and did not improve. This suggests that intervening on cognitive factors to improve ToM abilities doesn't lead to improvements in conversational ability or social interaction of this sort. Other interventions are possible. For example, early intervention to improve motor coordination could have any effect on conversational ability. I'll return to this possibility below.

Simply put, we may be able to identify factors relevant to a particular condition, and map them out using the model of a meshed architecture. Once identified, these are factors in the dynamical gestalt that we want to test by interventionist methods to discover whether and how such factors are causally related. An intervention on one factor (neuronal, cognitive, social, etc.) may shift things around, perhaps bestowing more weight on other factors, revealing causal relations that range from simple one-way causal relations to reciprocal, circular or non-linear dynamical relations. Contra the narrow view, an intervention can be more than just a manipulation of an internal mechanism (e.g., a drug effect on neuronal processes)—it can be a change in a environmental or social factor, or in bodily, or narrative practices, or, as may be the case in therapeutic contexts, some combination that can rearrange the entire gestalt.

### Coordination Dynamics

The third component of the methodological approach I am outlining is based on the work of Scott Kelso and colleagues on coordination dynamics. According to Kelso, “Dynamics is a language for connecting events from the genetic to the mental” (23). If we think of the motley collection of processes and factors that contribute to our human experiences, then the dynamics that define their relations may be the common element that allows us to explain how these processes are ordered or disordered. Although much of the work of Kelso and his colleagues focuses on brain dynamics, and they continue to frame their analysis in hierarchical and reductionist terms of top-down and bottom-up arrangements, their approach generalizes to any complex dynamical system and can apply to complex dynamical gestalts such as a biological, ecological or social systems that are typically bound together by coordination between dynamical processes operating at different spatio-temporal scales (66, 67). The measuring of coordination dynamics allows researchers to get into the fine details of dynamical processes and to develop explanatory models of how they are ordered. The method involves recording continuous time-varying processes as they unfold, and then analyzing the dynamical structure of such processes using time-series analysis. This allows for a more precise measuring of behavioral coordination by evaluating the synchronized patterning of system components as they change over time (2, 68).

Rather than tracking individual state variables, coordination dynamics studies “the topological features of the spatiotemporal patterns generated by virtue of their interaction” [(67), p. 2]. This type of analysis has focused mainly on rhythmic coordination, involving phase-locked synchronization and metastable coordination dynamics. In the latter case, phase relations are formed intermittently, rather than permanently, and this helps to preserve a diversity of processes (segregation) which are nonetheless linked (integration) (69). One can see this, for example, in social interaction, which requires both the autonomy of individual agents, and an interactional coupling that generates meaning (70). Different causal factors will have different weights in their relative contribution, some elements presumably, playing more important causal roles than others. We can think of the dynamical gestalt as a pattern, where, if one factor (or value or weight relative to the whole) is changed above a certain threshold, some or all of the other factors (and perhaps the whole) adjust. It is also in the dynamics that one will be able to measure different weighting patterns—including different connection weights between different factors [see, e.g., (71, 72)]. Such measurements will have significance for both explanation and for the tailoring of treatment.

Kelso and his colleagues are able to diagram meaningful changes in dynamical patterns as transitions in topological recurrence plots.

Overall, topological recurrence plots reveal local and global transitions of coordination patterns in the data that elude traditional methods…. Such irreducibility of certain topological properties may tap into the very nature of collective transitions in multiscale coordinative structures…. The relational quantities in such patterns constrain each other and form [more complex] structures which may not be discernible by examining each quantity individually…. [T]he ability of topological portraits to capture global properties is a key to detecting transitions in collective patterns that are not just an accumulation of pointwise changes…. In other words, topological portraits capture emergent features in the collective dynamics that are not reducible to the sum of its parts, tapping into a key feature of complex systems [(67), p. 10–11].

The patterns to be studied can be behavioral patterns [such as those involved in social interaction, e.g., (32, 73)], or neuronal activation patterns in the brain (74). This approach, which has focused on tracing the same dynamical principles across brain, body and social interaction, is meant to be integrative, not only revealing dynamical details in the meshing or integration among different variables, but providing an integrated theory of such patterns. This kind of analysis identifies “emergent phenomena where the whole is not only greater than the sum of its parts, but different too” (32).

The relevance of this method for the study of social interactions in the context of psychopathology, can be seen in the suggestion of Leong and Schilbach [(75), p. 636]: “disordered social interactions play a pervasive role in many, if not all, psychiatric disorders…. [T]hese disorders may result as much from an “interaction mismatch” across persons as from the breakdown of individual brains.” With respect to social cognition in specific, Dumas et al. (76) suggest that “the case of autism provides a test bed for an integrative approach.” Following this suggestion we can see how this 3-fold set of tools—the enhanced meshed architecture, experiments based on interventionist notions of causality, and the analysis of coordination dynamics—can help us explicate the dynamical processes characteristic of the gestalt of factors involved in ASD.

## Autism as a Test Case

If the model of a meshed architecture can identify which processes or factors to ask about, an interventionist strategy to test for causal or constitutive relevance can show us how factors are actually meshing, and the application of coordination dynamics can fill in some of the details of the dynamics to give us a more complete model. Let's consider ASD as a test case for this sort of analysis.

### ToM and Social Cognition

Theoretical and empirical considerations suggest multiple relevant factors in regard to questions about ASD. Compared to typically developing children, one of the primary differences found in children with ASD involves social cognition and intersubjective interaction. A standard approach is to consider such problems in social cognition to involve deficits in theory of mind (ToM), specifically in abilities to “mindread,” i.e., to make theoretical inferences about one's own or another person's mental states (77, 78), or to simulate or empathize, due perhaps to disruption in the activation of the mirror neuron system (79, 80).

Such views are informed by empirical studies that show children with autism fail to pass false-belief tasks (77, 81). In the classic experiments, typically developing (TD) children at 4 years of age, on average, but not children of <4 years, are able to distinguish between how things really are in the world and what other people may falsely believe about such things. Around this age [if not earlier—see (82)], we begin to recognize that other individuals have their own sets of beliefs and intentions that inform their behavior, and we are able to explain or predict their behavior based on these mental states. Significantly, however, individuals with ASD fail false-belief tests even at mental ages significantly higher than 4 years. Individuals with autism are thus said to lack a theory of mind, and this cognitive deficit explains their lack of social responsiveness and understanding. Such views informed the experiments by Hadwin et al. (65), discussed above. In those experiments the intervention targeted specific cognitive abilities associated with ToM, but, as noted, failed to improve social communicative interaction.

### ASD and Affectivity

According to ToM approaches, the important factors are cognitive and intersubjective processes. It is likely, however, that other factors are involved. Hobson (83), for example, has argued that affective factors are directly relevant in ASD. He cites empirical developmental studies that show the importance of affective relatedness in the infants' developing capacities for social interaction as early as the 1st year of life: “very young infants have perceptual-affective responsiveness to some aspects of another person's affect-related expressiveness and behavior, even though they may not be able to discriminate specific “meanings” in abstracted expressions until the middle of their 1st year” [(83), p. 232]. Affective relationality is, according to Hobson, the basis for primary intersubjectivity (84) and manifests itself both behaviorally and experientially. He argues that deficits in affective relationality, and its concomitant effect on intersubjective interactions, lead to the cognitive difficulties seen in ASD, rather than the other way around [(83), p. 227; see (85)].

This speaks to both the integrated relations of different factors (cognitive and affective), and a certain order in the meshed architecture. In working out the meshed relations, however, one does not need to take a deficit in affective relationality to be the core or generative deficit in autism (86). More generally, taking ASD to involve a particular disorder means that there may not be any one deficit (whether cognitive, affective, or social) that can be identified as the core deficit [see (87)]. Rather, we can think that ASD always involves a pattern of differences from typical development, such that it is enough to maintain that problems with affective relationality may be part of the overall pattern in ASD. Indeed, Bird and Cook (88), acknowledging that ASD may be associated with disordered emotion processing and deficits of emotional reciprocity, argue that there are wide variations in this regard across the spectrum. They suggest that when deficits in affective relationality are observed, they may be due to a frequently co-occuring alexithymia (a condition involving reduced ability to identify or describe one's own emotion, resulting in reduced empathy and impaired ability to recognize emotions in others), rather than being a feature of autism itself. It may be alexithymia that interferes with the passing of false-belief tasks if the task involves identifying an emotion. Bird and Cook (88) suggest that the complication with alexithymia means that social impairments may be distinct from emotional impairments in the case of ASD. Although this is not necessarily the accepted view [see (89, 90)], there is growing evidence that supports the idea that difficulties in emotion processing, and lower accuracy with facial emotion recognition in people with autism, are due to alexithymia (91–93). Further research on this would require identifying individuals with autism who manifest problems with emotion processing, intersubjectivity and social communication more generally, but who do not have alexithymia.

### Motor Control

Whether due to ASD or alexithymia, impairments in the production and recognition of bodily affective expressions, and in the communication of feelings, broaden to communicative behavior generally, including problems with gesture. Thus, individuals with autism “rarely make gestures such as showing, giving or pointing in order to share awareness of an object's existence or properties, or comprehend such gestures when they are made by others….” [(83), p. 242]. Klin et al. (94), following an enactive model, show that individuals with autism fail to follow another's pointing gesture in some cases, and this may be tied to a lack of expertise in social perception, tied to differences in visual focus when viewing complex social situation. Their experiments show that individuals with autism direct their gaze to aspects of the environment, or to other people, in ways that miss socially salient information, for example, focusing on a person's chin instead of on their eyes.

This may also be linked to evidence that in ASD, besides cognitive and affective factors, there is some deficit in bodily processes that involve sensory-motor performance. Studies of individuals with ASD show that sensory–motor problems (specifically disrupted patterns in afferent and proprioceptive sensory feedback) can interfere with motor control (95–97). These may involve postural instabilities, atypical gait, mistiming of motor sequences, motor coordination problems, problems with anticipatory postural adjustments and expressionless faces (98–103), all of which involve aspects that normally scaffold effective social interactions and thus have implications for primary intersubjectivity (104, 105). For example, some studies have shown that children with ASD have difficulties in predicting *why* an action is being done by another person based on kinematic cues and this may reflect difficulties in the children's own motor planning [(98, 106, 107)].

Multiple studies show that infants at heightened risk and later diagnosed with ASD at 36 months have a slower progression in the development of unsupported sitting and walking (108, 109), as well as grasping and functional object use (110). Ability for grasping and manipulating objects is important for sharing them with others, and has been shown to support communicative ability and word learning in typical development (111, 112). Indeed, differential development of motor skills may have cascading effects on vocabulary acquisition, gestures and social skills, which suggests a different account of the origins of problems in social interactions compared to traditional ToM approaches (110, 113, 114).

### Vertical and Horizontal Meshing

Iverson and Wozniack (115) suggest that communicative delays and atypicalities in intentional and symbolic communication in children with ASD should be considered as extending beyond the individual since they have an impact on social partners and the communicative environment understood in a broader sense. In terms of the enhanced meshed architecture described above, this would involve the horizontal axis, and a context-related dynamic interplay between the communicator and his/her social and material environment, including artifacts, instruments, and established practices in communicating with other people. Likewise, the intrinsic dynamics that involve motor control and affectivity, on the vertical axis, include the development of habits and basic skills, which, may be relevant to social communication, and are often important for participating in joint action and for processes involved in intersubjective interaction.

In contrast to the study by Hadwin et al. (65) on deficits in theory of mind (ToM) development, discussed above, an experiment by García-Pérez et al. (116) gets us closer to what may be some more basic problems in the conversational ability of autistic children, and points to a tight meshing of affect, motor control processes, intersubjectivity and factors that define the horizontal axis [see (117, 118)]. The experiment on conversational ability showed that subjects with ASD “made fewer headshakes/nods (but not smiles) when the interviewer was talking” (p. 1310).^3^ A principle of social interaction, reflected in detailed analyzes of the distributed semiotics of conversational processes [e.g., (120)], is that it involves two-way, context-rich interaction. In this respect García-Pérez et al. also studied the responses of the conversational partners, the interviewers who interacted with the children with ASD. It turns out that they also made fewer head-shakes/nods when the children were talking compared to their typical conversation style. In this regard the dynamics of the conversation itself, as a whole, change. This kind of two-sided disruption was reflected in significant differences measured as “subjective” ratings of (a) affective engagement and (b) the smoothness of reciprocal interaction (as rated by two evaluators blind to diagnosis, rating emotional connection during the videotaped conversation and the flow of the conversation on scales of 1–5).

García-Pérez et al. suggest that children with ASD show a deficient propensity to engage with the bodily-expressed attitudes of others [also see (121)]. It's important, however, not to lose track of the other side of the conversation. It's not only the children with ASD that show a deficient propensity to engage with the bodily-expressed attitudes of others, those in conversation with the children do so as well. The reciprocal dynamics that constitute the conversation collapse on both sides. As McGeer (122) notes, the burden of understanding is typically distributed between the participants who are trying to understand each other. Failure on one side may be reinforced by failure on the other side.

In the case of ASD, disruptions seemingly occur along both axes of the meshed architecture. On the vertical axis the verbal accomplishment of thought seems impaired in conversation when most responses are simple answer-type replies. This impoverishment of expressed thought may not be divorced from the various anomalies involved in sensory-motor processes that can disrupt the intrinsic control processes involved in non-verbal and gestural performance (94). Likewise, problems in the affective dimension may disrupt the possibility of smooth dynamics amongst these factors as well as with factors on the horizontal axis. The deficient interactive response of others to people with ASD may not only reinforce disruptions to communicative processes, but may also signal a change in normative expectations that accompany most social and cultural practices. Indeed, the integrative meshing of what we called the whole dynamical gestalt of intersubjective interaction seems to be differently aligned in the case of ASD [see (49), for more detail].

It's important to note that social impairments in autism are not limited to disruptions of real time coordination dynamics (as one reviewer has pointed out). They can also involve a failure of people with autism to comfortably inhabit built environments and social institutions that are organized to accommodate neurotypical styles of engagement. It's not just about eye contact or the timing of conversational responses (which may involve processes in a very short time scale—sometimes measured in seconds), but it may also include the amount of lighting or background music used in public spaces, social arrangements or cultural practices that may be upsetting to some individuals with ASD, or even disruptive to development (on a longer time scale that may be measured in years). Negotiating such material and social/normative, neurotypical environments may have a long-term impact on their embodied interactions with others within these spaces, and their “habits of mind” (123), or ways of being in the world. It may lead to what are sometimes observed in ASD, namely, “highly structured and regimented life routines that avoid novelty and the inherent unpredictability of typical social life” [(94), p. 345]. Similar things can be said about what Constant et al. [(7), p. 7] call “practical causality” (or looping effects) in the medical or psychiatric context—that is, changes in behavior caused by virtue of being classified or labeled autistic. Classifying someone as autistic may in fact change expectations and behavior.

### Interventions

Intervention into this complex gestalt of different factors can happen in numerous ways. We saw a clear, albeit unsuccessful, but nonetheless telling example in training on ToM tasks (65). Psychological therapies that target emotional awareness or provide emotion recognition training, however, may improve social communication in ASD (124, 125). It is also possible to intervene by training motor control processes *via* procedural learning (107, 126). Moreover, one could think that intervention would be possible by changing the behavior of the conversational interlocutors. Importantly, it may be that improved therapy requires interventions on multiple factors.

Consider, for example, a study by Kostrubiec et al. (127) (referencing Kelso's coordination dynamics approach). The researchers compared 20 ASD vs. 21 typically developing (8–14 years of age) children on motor coordination in intentional motor tasks. They correlated the results to Socio-Adaptatif Quotient (SAQ) scores that measure communication ability and socialization (e.g., saying “please,” “thanks,” or “excuse me”). They found no notable differences in a simple, perceptual-motor coordination task; but children with ASD showed increasing deficits in more demanding interpersonal coordination tasks when coordination patterns were intentionally requested by the experimenter [consistent with other studies—e.g., (68, 128)]. This result also correlated to poor SAQ scores. Improved performance in older ASD children, however, suggested that therapeutic intervention would be possible. This led the researchers to the following hypothesis: “by manipulating [motor coordination] parameters in ASD children, their coordination abnormalities could be reduced, and their social deficits, perhaps, alleviated” (127). Things are more complicated, however, since there were increased deficits in coordination when socially interacting with the experimenters, and since in other disorders, e.g., ADHD, anomalies in motor coordination do not lead to similar social deficits. This suggests not a one-way or one-to-one causal relation, but some more nuanced (dynamical, non-linear) relation between social interaction and motor coordination.

The therapeutic intervention might also be based on considering the communicative practices of the experimenters as they interact with children with ASD. Context is also surely important, and communicative interactions may be very different outside the lab, in the home, when, for example, children with ASD are interacting with parents [see (129) for an insightful ethnographic study]. Indeed, considering that there may be deficiencies in several factors, the reference to Kelso's coordination dynamics approach is apropos.

### The Dynamics of ASD

Embodied and enactive perspectives on social cognition emphasize the importance of movement coordination of one's body with the other person while performing actions (social-motor coordination) (130–133). As noted in previous sections, children with ASD have difficulties in social communication skills, some of which may be due to motor deficits (found in 50–80% of children diagnosed with ASD) (134–136).

Fitzpatrick et al. (137) suggest:

Social motor coordination both in the form of imitation and in the lesser known phenomenon of interactional synchrony, is important for maintaining critical aspects of successful human social interaction, including interpersonal responsiveness, social rapport and other-directedness… positive self-other relations… and verbal communication and comprehension (137).

Social synchronization is an important component of interpersonal interaction, across a wide diversity of situations, including emotional arousal, imitation, joint attention, parent–child exchanges, mutual gaze, shared attention, and empathy (138, 139). A breakdown or significant modulation of such synchronization or entrainment across many of these contexts are reported in children and adolescents with ASD [e.g., (140–142)]. In the case of stable coordination patterns, the variability of relative phasing (the phase relation between movement patterns) between two or more individuals reflects the strength of coupling or alignment. Higher variability, as found in ASD, reflects weaker coupling.

Using coupled oscillator modeling,^4^ Fitzpatrick et al. (68) showed that adolescents with ASD demonstrate less interactive synchronization in both spontaneous and intentional social coordination, corresponding to lower sensitivity and decreased attention to the other person.

Adolescents with ASD demonstrated a disruption of both spontaneous synchronization and intentional synchronization…. [T]he ASD group [compared to typically developing adolescents] had weaker spontaneous synchronization … when participants were viewing each other's pendulum…. ASD participants synchronized less well under conditions in which synchronization occurs spontaneously in the presence of perceptual information of the social partner and in situations when there is an explicit social goal to coordinate with another person (e.g., intentional synchronization).

The researchers suggest two possible interpretations of these results, namely that the synchronization problems of adolescents with ASD involved problems with either attention or motor control. Di Cesare et al. (143) suggest that it may also involve lack of perceptual sensitivity to variation in vitality forms, but that this too may be due to motor atypicalities in ASD [also see (68, 135)].

An alternative method using coordination dynamics to study non-linear dynamics of human social coordination, including behavioral measures of social interaction (and interpersonal synchrony) in people with ASD, employs a research tool called the Human Dynamic Clamp (HDC). This set-up allows a dynamic bidirectional interaction in real time between a human and a virtual avatar (modeled on human-human interaction) (76, 144). Using this method, Baillin et al. (136) studied behavioral aspects of interpersonal synchrony in ASD, considering several variables, including motor control and emotion recognition.

Noting that children with ASD have difficulties in coordinating their body during social exchange (see the research cited above), Baillin et al. showed lower motor skills among ASD participants suggesting a significant link between motor skills and social-cognitive skills among a population of children and adolescents. The low score on motor ability was the only significant factor that distinguished the ASD from the typically developing individuals.

Social interaction is not unidirectional, of course. We know that there is a difference between a one-way coupling, for example when a participant is responding to the movement of a computer-generated avatar who is not responding to them, and a two-way interaction [see (145)]. As indicated above, one's own response may depend on how one's interlocutor or social partner responds. The use of coordination dynamics should also be able to throw light on this. The degree to which social partners contribute to entrainment in joint coordination may differ. The results of the above experiments suggest that persons with autism may adapt their movements less to those of their partner. But it is also possible that (1) the social partner compensates for this by adjusting their own movement, or (2) that the partner shows less entrained attunement when coordinating with an individual with ASD.^5^ There is some evidence of differences in people's motoric response to those they perceive as psychologically different. For example, Brezis et al. (148) report that an experimenter, who was not blind to the diagnosis, interacting with subjects with ASD, moved more slowly than when interacting with typically developing participants [also see (149)]. This is a question that calls for further experimentation and clarification. Generally speaking, interaction is reciprocal in the sense that in typical social encounters understanding is distributed between the person trying to understand and the person who is trying to make herself understandable (122).

To conclude, these studies of interactional dynamics in ASD can yield significant detail about the precise nature of disordered communication and interactive practices. They clearly motivate further experiments and more comprehensive therapeutic interventions that can help to define the dynamical relations existing among motoric, affective, cognitive, social and ecological factors and their disruption in ASD [see (150, 151) for just such a comprehensive approach]. This, in turn, would allow us to map more precisely the meshed architecture of the dynamical gestalt in the context of social interaction and relevant disruptions.

## Conclusion

I set out to address the integration problem in psychiatry. I noted that this problem is tied to conceptions of causality and explanatory levels in our understanding of mind and human social existence more generally. I proposed a 3-fold method for exploring the dynamics of integration, based on a concept of dynamical causation and a non-hierarchical (level-free) notion of gestalt. In these terms I've explored ASD as a test case. This approach supports an analysis that both distinguishes and integrates the various factors and processes that contribute to differences in social engagement found in ASD. The model is different from, on the one hand, most standard explanations of autism in terms of ToM or any one factor. It rather focuses on a pattern of dynamically intertwined factors. On the other hand, this model is also different from the model of a fused integration (1), which would necessarily fail to capture or discriminate the specific factors that contribute to this pattern.

On this dynamical gestalt view I've argued:

1. We can give up explanatory concepts of levels or hierarchy;
2. but still use interventionist analysis to identify causal relevance—non-linear/dynamical causality that allows useful distinctions to be made between various factors/processes, and at the same time, can account for their integration;
3. and provide a dynamical systems explanation that can sort out in some detail the dynamical relations that define the gestalt.

I take this approach to be consistent with a wide enactive approach to psychiatry which offers alternatives to reductionist explanations in terms of hierarchical levels [see (1)] and emphasizes the dynamical interaction of factors that span brain-body-(physical and social) environment.

## Data Availability Statement

The original contributions presented in the study are included in the article/supplementary material, further inquiries can be directed to the corresponding author.

## Author Contributions

The author confirms being the sole contributor of this work and has approved it for publication.

## Funding

This research was supported by the Lillian and Morrie Moss Professor of Excellence in Philosophy Endowment. SG completed the finalized version of this paper thanks to a funded position as Visiting Professor of Psychology, University of Rome—Sapienza.

## Conflict of Interest

The author declares that the research was conducted in the absence of any commercial or financial relationships that could be construed as a potential conflict of interest.

## Publisher's Note

All claims expressed in this article are solely those of the authors and do not necessarily represent those of their affiliated organizations, or those of the publisher, the editors and the reviewers. Any product that may be evaluated in this article, or claim that may be made by its manufacturer, is not guaranteed or endorsed by the publisher.

## Footnotes

^1^The term “narrow” (vs. “wide”) is a term of art in philosophy of mind, signifying cognitive processes that are in the intra-cranial mind, the brain, or “in the head.” “Wide” signifies the involvement of extra-neural, embodied and ecological processes. To be clear, my concern is to address a wide conception of integration which is not reducible to the kind of neural integration that one finds, e.g., in Panksepp and Northoff (8).

^2^This is a pragmatic objection about the use of the notion of emergence as fusion in psychiatric contexts. There is more to say about the theoretical explanation of emergence as fusion as found in Humphreys and de Haan. As one reviewer points out, the concept of levels may indeed be implicit in the concept of emergence, and even explicit in the explanation of emergence as Humphreys and de Haan use it. Humphreys, for example, as evident in the quotation above, still refers to higher-level and lower-level properties. It is also implicit in the standard concept of gestalt, where one might consider the whole to be at a higher level than the parts. I agree there is something misleading about this. One might argue that if the ingredients or properties “at the lower level” go out of existence, then there is no lower level—it gets subsumed into the “higher level”—one is left with a whole without parts, or a system where the level distinction is not relevant. This is de Haan's analogy with the cake. It's not clear that the ingredients are still there—they have been chemically transformed as they are fused into the cake. Humphreys indicates that the lower-level property instances go out of existence. The causal properties of the emerged entity are not the same as the causal properties of the original property instances, which no longer exist or have independent existence within the fusion. Despite no longer existing, de Haan continues to refer to parts that make up the whole.

^3^Although a study of adolescents with ASD conducted by Capps et al. (119) found more headshaking and nodding in response to yes or no questions, the subjects were less likely to nod while listening to their conversational partners talk. They concluded that “children with ASD demonstrated limited involvement in the co-construction of a shared conversational trajectory through non-verbal as well as verbal channels” [(119). p. 337].

^4^This task allows for the study of both intentional (following instruction) and spontaneous interpersonal coordination. Two people coordinate handheld pendulums swinging them “from the wrist joint in the sagittal plane (using radial-ulnar abduction–adduction). This methodology has demonstrated that the strength of interpersonal synchronization … can be understood in terms of a dynamical model of synchronization…. Using such a dynamical model to understand how synchrony breaks down in social deficits has the distinct advantage of allowing one to infer which dynamical components of the model are underlying the impairment” [(137), p. 3; also see (128)].

^5^Peper et al. (146) point to a possible strategy to measure these differences developed by Słowiński et al. (147).
